# Activity of acetyltransferase toxins involved in *Salmonella* persister formation during macrophage infection

**DOI:** 10.1038/s41467-018-04472-6

**Published:** 2018-05-18

**Authors:** Julian A. Rycroft, Bridget Gollan, Grzegorz J. Grabe, Alexander Hall, Angela M. Cheverton, Gerald Larrouy-Maumus, Stephen A. Hare, Sophie Helaine

**Affiliations:** 1 Section of Microbiology, Medical Research Council Centre for Molecular Bacteriology and Infection, Imperial College London, Armstrong Road, London, SW7 2AZ UK; 2 Department of Life Sciences, Medical Research Council Centre for Molecular Bacteriology and Infection, Imperial College London, Armstrong Road, London, SW7 2AZ UK; 3 Department of Life Sciences, Imperial College London, Exhibition Road, London, SW7 2AZ UK; 4 School of Life Sciences, University of Sussex, Brighton, BN1 9QG UK

## Abstract

Non-typhoidal *Salmonella* strains are responsible for invasive infections associated with high mortality and recurrence in sub-Saharan Africa, and there is strong evidence for clonal relapse following antibiotic treatment. Persisters are non-growing bacteria that are thought to be responsible for the recalcitrance of many infections to antibiotics. Toxin–antitoxin systems are stress-responsive elements that are important for *Salmonella* persister formation, specifically during infection. Here, we report the analysis of persister formation of clinical invasive strains of *Salmonella* Typhimurium and Enteritidis in human primary macrophages. We show that all the invasive clinical isolates of both serovars that we tested produce high levels of persisters following internalization by human macrophages. Our genome comparison reveals that *S*. Enteritidis and *S*. Typhimurium strains contain three acetyltransferase toxins that we characterize structurally and functionally. We show that all induce the persister state by inhibiting translation through acetylation of aminoacyl-tRNAs. However, they differ in their potency and target partially different subsets of aminoacyl-tRNAs, potentially accounting for their non-redundant effect.

## Abstract (web summary)

The recalcitrance of many infections to antibiotic treatment may be due to the presence of ‘persisters’, or non-growing, antibiotic-tolerant bacteria. Here, the authors study the structures and functions of aminoacyl-tRNA acetyltransferase toxins, and their roles in persister formation in *Salmonella*.

## Introduction

Non-typhoidal *Salmonella*e (NTS), mostly *Salmonella enterica* serovars Typhimurium and Enteritidis, cause localized intestinal infection in immunocompetent humans, and invasive non-typhoidal disease (iNTS) in HIV-infected adults (where NTS serovars comprise over a third of all bacteraemias^1^) and in young children under 5 years old^2–4^. It is estimated that 3.4 million cases of iNTS occur globally each year^5, 6^, primarily in sub-Saharan Africa. Although the global incidence of iNTS is much less than typhoid fever, the associated mortality rate of 20–25% in immunocompetent individuals can exceed 50% in HIV-infected patients^3, 7, 8^. This suggests that iNTS kills three- to fourfold more people than typhoid, with estimated deaths per annum approaching 700,000^5, 6^.

Cephalosporins remain the first-line antibiotic treatment in Africa and in Europe, but invasive strains are almost universally resistant to the first- and second-generation options. Increasing reports of resistance to third-generation cephalosporins^9^ have bleak implications. Recurrence of the disease has been observed in 20–43% of cases, which is attributable to reinfection with distinct bacterial isolates only in a minority of cases (22%)^10^. The remaining 78% represent relapses with the same strain. Although this may result from chronic septic foci following incomplete antibiotic treatment or poor drug penetrance to the infected tissues, there is a growing body of evidence indicating this could be due to growth resuscitation of intracellular persister bacteria which, after a period of transient antibiotic-tolerant growth arrest, resume proliferation resulting in clonal relapse of infection^10–14^.

*Salmonella* Typhimurium 12023/14028 is a commonly used laboratory strain originally obtained from cattle. We reported previously that after phagocytosis of this strain by murine macrophages, a significant proportion of the population ceases to grow and is composed of antibiotic-tolerant persisters^11^. We have found that a repertoire of class II toxin/antitoxin (TA) modules, although having no impact on stochastic persister formation in the laboratory medium, is specifically induced upon uptake of the bacteria by macrophages. In infection conditions, these stress-responsive elements are involved in the formation of intramacrophage persisters in a non-redundant manner^11^. Class II TA operons encode a non-secreted toxin, which inhibits an essential cellular function such as RNA translation or DNA replication, and an antitoxin that interacts with and neutralizes the toxin^15^. The toxin is relatively stable, whereas the antitoxin is labile and degraded under various stress conditions, leading to the build-up of free toxin and growth arrest of the bacterial cell^16^. We recently revealed that one such toxin, TacT, contributes to *Salmonella* Typhimurium entry into the persister state through acetylation of aminoacyl-tRNA molecules, thereby halting translation^17^.

Here we investigate whether strains of *S*. Typhimurium and *S*. Enteritidis isolated from humans also respond to cellular internalization by a 1000-fold increase in persister formation. We characterize persister induction in a collection of seven clinical isolates from patients in Malawi and find that all the invasive strains of *S*. Typhimurium and Enteritidis that we tested form persisters to a similar extent following internalization by human macrophages. A genomic comparison reveals that all strains encode three TacT-like toxins amongst a conserved repertoire of TAs. We fully characterize the structure and function of all the related Tac toxins found in *S*. Enteritidis and *S*. Typhimurium. We show that all three toxins induce the persister state by inhibiting translation through acetylation of aminoacyl-tRNAs. However, they differ in their potency and target partially different subsets of aminoacyl-tRNAs, potentially accounting for their non-redundant effect.

## Results

### Non-typhoidal *Salmonella* form persisters in human macrophages

**Fig. 1:**
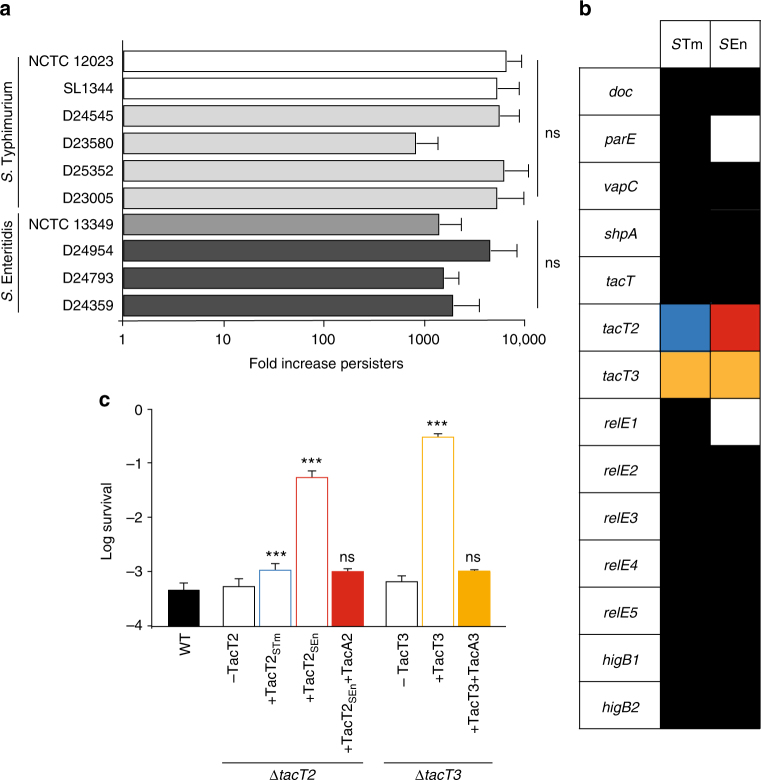
Non-typhoidal *Salmonella* strains induce persister formation following internalization by human macrophages. **a** Fold increase in persisters caused by 30 min internalization in primary human macrophages relative to levels in inocula in clinical isolates of *S*. Typhimurium (white and light grey bars) and *S*. Enteritidis (dark grey bars), measured after exposure to gentamicin. Data represent the mean ± SEM (*n* ≥ 5) and were analysed using one-way ANOVA. **b** Comparison of the repertoire of class II TA modules between *S*. Typhimurium and *S*. Enteritidis serovars. Boxes of same colour—fully conserved; white boxes—absence; different colour boxes—polymorphism. **c** Proportion of bacteria surviving 4 h exposure to bactericidal concentrations of cefotaxime in cultures of *S*. Typhimurium 12023 wild-type, Δ*tacAT2*, Δ*tacAT2* pBAD33::*tacT2*_*STm*_, Δ*tacAT2* pBAD33::*tacT2*_*SEn*_, Δ*tacAT2* pBAD33::*tacT2*_*SEn*_ and pCA24N::*tacA2*, or Δ*tacAT3*, Δ*tacAT3* pBAD33::*tacT3*, Δ*tacAT3* pBAD33::*tacT3* and pCA24N::*tacA3*. Arabinose and IPTG were added to all cultures in the fresh medium during the lag phase, then antibiotic treatment started 1 h later. Data represent the mean ± SEM (*n* ≥ 3) and were analysed using a Student’s *t* test, compared to WT (ns, non-significant; ***p < 0.005)

We reported that the formation of persisters in the laboratory reference strain of *S*. Typhimurium 12023 is dramatically increased after phagocytosis by murine macrophages^11^. Because of the strong evidence for clonal relapse of *Salmonella* infection following antibiotic treatment^10^, we investigated persister formation of clinical invasive strains of *S*. Typhimurium and Enteritidis (four and three isolates, respectively) in human primary macrophages. All clinical isolates were collected from blood cultures taken from patients in the Queen Elizabeth Hospital, Blantyre in Malawi between 2003–04 (Supplementary Table 1). We compared the behaviour of each clinical isolate to that of the broadly studied reference stains of each serovar. For all clinical and reference isolates, internalization of *Salmonella* by human macrophages stimulated a similar increase (of approximately 1000-fold) in the proportion of persisters, compared to those already present in the inoculum (Fig. 1a), with similar proportions being recovered after exposure to gentamicin (all Typhimurium strains: log percentage survival −1.65 ± 0.23) or cefotaxime (log percentage survival −1.19 ± 0.3). Very similar stimulation of persister formation was observed for all the isolates after uptake by macrophage-like cell lines, as evidenced by the biphasic killing curves (Supplementary Fig. 1). This experiment reveals that the formation of antibiotic-tolerant persisters in response to internalization by host cells is a common feature of the *Salmonella* strains.

Fourteen chromosomal class II TA modules were identified previously in *S*. Typhimurium 12023 as contributing to persister formation^11^. We compared the repertoire of the class II TA modules in the different clinical isolates. Repertoires are perfectly conserved between the strains of the same serovar, and the comparison between *S*. Typhimurium and *S*. Enteritidis revealed that all *S*. Enteritidis isolates display a one amino acid change in one of the toxins, and that they also lack *relBE1* and *parDE* (Fig. 1b) that are important TA modules for the formation of persisters by *S*. Typhimurium^11^. The polymorphic toxin was an uncharacterized toxin sharing significant amino acid identity (46.8%) with the previously characterized TacT (Supplementary Fig. 2); we named it TacT2. A third-related toxin, TacT3, showing 28.3% of the amino acid identity to TacT, is conserved across serovars (Supplementary Fig. 2). Since no significant difference was noticeable in the ability of strains from the two serovars to form persisters (Fig. 1a), we hypothesized that TacT2_SEn_ might compensate for the absence of RelE1 and ParE in persister formation.

Growth arrest induced by the activity of TA toxins stimulates the formation of antibiotic-tolerant persister bacteria. Accordingly, overexpression of *tacT2*_*SEn*_ or *tacT3* and to a lesser extent *tacT2*_*STm*_ into the single deletion mutant of the corresponding TA module led to an increase in the proportion of *Salmonella* surviving the exposure to bactericidal concentrations of cefotaxime during growth in vitro, without having affected the MIC (Fig. 1c and Supplementary Fig. 3A). This corresponded to an increase in persister proportions, as illustrated by the biphasic killing curves obtained upon exposure to 20 times the MIC, out of the range where survival rate is influenced by the antibiotic dose (Supplementary Fig. 3B). The increase in persister formation induced by overexpression of *tacT2*_*SEn*_ and *tacT3* was fully counteracted by overexpression of their respective antitoxins, *tacA2* and *tacA3* (Fig. 1c). Together, these results indicate that TacT2_SEn_ promotes persister formation to a greater extent with respect to TacT2_STm_.

### Acetyltransferases TacT2_SEn_ and TacT3 alter the translation

We previously showed that TacT is a Gcn5 *N*-acetyltransferase (GNAT) toxin that controls *Salmonella* growth through acetylation of aminoacyl-tRNA molecules^17^.

**Fig. 2:**
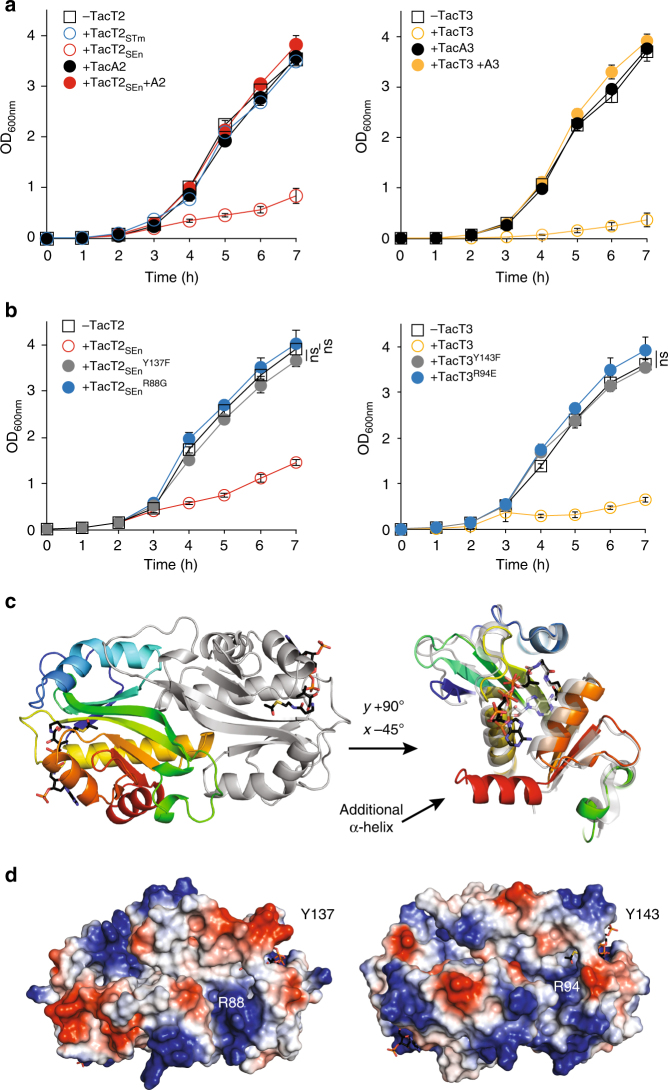
TacT2_SEn_ and TacT3 are functional acetyltransferase toxins. **a** Left panel: Growth curves of *S*. Typhimurium 12023 Δ*tacTA2* carrying pBAD33 (−TacT2), pBAD33::*tacT2*_*STm*_ (+TacT2_STm_), pBAD33::*tacT2*_*SEn*_ (+TacT2_SEn_), pCA24N::*TacA2* (+TacA2) or pBAD33::*tacT2*_*SEn*_ and pCA24N::*tacA2* (+TacT2_SEn_ + TacA2). Right panel: Growth curves of *S*. Typhimurium 12023 Δ*tacTA3* carrying pBAD33 (−TacT3), pBAD33::*tacT3* (+TacT3), pCA24N::*TacA3* (+TacA3) or pBAD33::*tacT3* and pCA24N::*tacA3* (+TacT3 + TacA3). All cultures were supplemented with arabinose and IPTG in fresh rich medium during the lag phase and growth was monitored by optical density. **b** Left panel: Growth curves of *S*. Typhimurium 12023 Δ*tacTA2* expressing from pBAD33, the wild-type *S*. Enteritidis toxin (+TacT2_SEn_) or point mutant toxins (+TacT2^Y137F^_SEn_) or (+TacT2^R88G^_SEn_), or carrying the empty vector (−TacT2). Right panel: Growth curves of *S*. Typhimurium 12023 Δ*tacTA3* expressing from pBAD33, the wild-type toxin (+TacT3), or point mutant toxins (+TacT3^Y143F^) or (+TacT3^R94E^), or carrying the empty vector (−TacT3). All cultures were supplemented with arabinose in fresh rich medium during the lag phase. **c** Left panel: Cartoon representation of the dimeric structure of TacT3^Y143F^. Chain A is coloured from blue at the N terminus to red at the C terminus, and Chain B is coloured light grey. Right panel: Cartoon superimposition of TacT3^Y143F^ (coloured) and TacT^Y140F^ (grey). TacT3- and TacT-bound Ac-CoA molecules are shown as black and grey sticks, respectively. (PDB: 6G96). **d** Electrostatic potential of the surface of the modelled TacT2_SEn_ (left panel) or TacT3 (right panel) dimer showing positive potential in blue and negative in red. Residues mentioned in the text are labelled. **a**, **b** Data represent the mean ± SEM (*n* ≥ 3) and were analysed using a Student’s *t* test (ns, non-significant)

Overexpression of the *tacT2*_*STm*_ gene in a deletion mutant of the corresponding TA module had no noticeable effect on *S*. Typhimurium growth, whereas overexpression of *tacT2*_*SEn*_ extended the lag phase dramatically (Fig. 2a and Supplementary Fig. 4). Overexpression of the *tacT3* gene in a deletion mutant of the corresponding TA module dramatically extended the lag phase of *S*. Typhimurium (Fig. 2a and Supplementary Fig. 4), indicating that TacT2_SEn_ and TacT3, but not TacT2_STm_ toxins, prolong the bacterial non-growing state. This growth inhibition was counteracted by concomitant overexpression of the corresponding antitoxin *tacA2* or *tacA3* gene (Fig. 2a). These results show that the TacAT2_SEn_ and TacAT3 operons encode functional TA modules.

The toxicity of TacT is dependent on its acetyltransferase activity (the transfer of an acetyl group from acetyl coenzyme A (Ac-CoA) to the target molecule). Accordingly, for each toxin, a single amino acid substitution of the predicted catalytic site responsible for transfer of the acetyl moiety to the acceptor molecule (Y137F for TacT2_SEn_ and Y143F for TacT3) completely abolished the toxicity in *Salmonella* (Fig. 2b).

The crystal structure of the purified non-toxic TacT^Y140F^ mutant protein revealed an acetyltransferase fold and an Ac-CoA molecule associated in the active site^17^. We obtained the crystal structure of purified TacT3^Y143F^ in 1.5 Å resolution (Fig. 2c–d and Supplementary Fig. 5, and Supplementary Table 3). The fold of TacT3 appeared to be that of an acetyltransferase similar to TacT. with the exception of an additional C-terminal alpha-helix (Fig. 2c, right panel). Based on its high identity with TacT (46.8%), we modelled the structure of TacT2 (Fig. 2d, right panel). Similarly to our results with TacT, purified TacT3 forms a dimer with a comparable dimerization interface (Supplementary Fig. 6). Examination of the surface electrostatic potential of TacT2 and TacT3 revealed a positively charged groove, leading from the active site of one monomer to another patch of positive charge on the second molecule of the dimer (Fig. 2d)—features also present at the surface of TacT. Single amino acid substitution of R88G and R94E in TacT2_SEn_ and TacT3, respectively, abolished the toxicity in *Salmonella*, in agreement with our previous data for TacT (Fig. 2b). These results show that, similarly to TacT, TacT2_SEn_ and TacT3 exhibit positive amino acids at their surface that are essential for their activity.

TacT promotes persister formation through interference with protein synthesis^17^. To determine if TacT2_SEn_ and TacT3 have the same activity, we measured the rates of translation using pulse-chase of radio-labelled methionine^18^. As a positive control, we treated a bacterial culture with chloramphenicol, as this antibiotic inhibits mRNA translation. Upon induction of gene expression of *tacT2*_*SEn*_ and *tacT3*, but not of the *parE* toxin predicted to target DNA, methionine incorporation was immediately decreased (Supplementary Fig. 7), indicating that all active TacT toxins alter translation.

### Tac toxins block translation through aminoacyl-tRNA acetylation

**Fig. 3:**
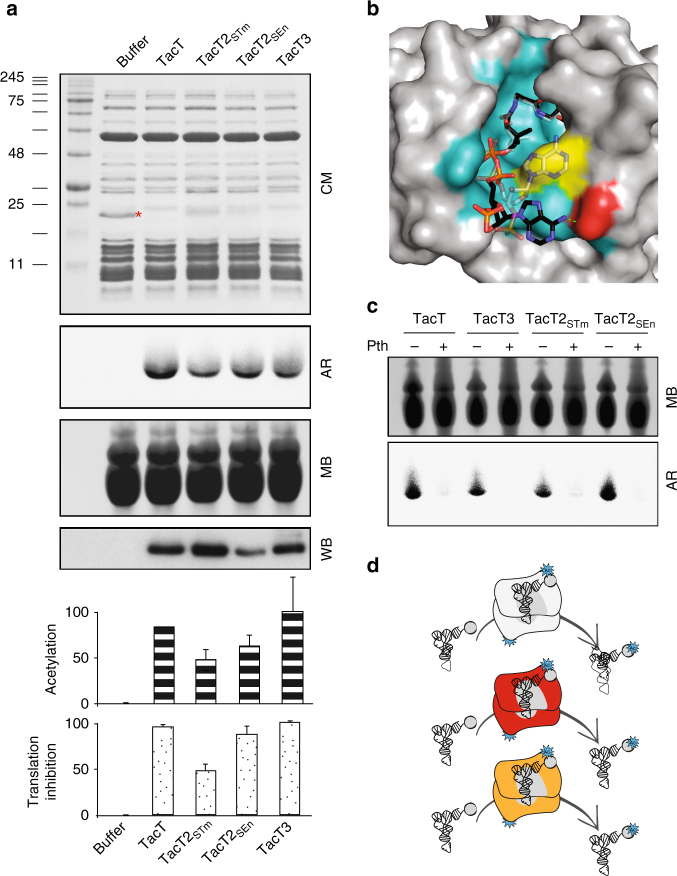
Tac toxins block translation through acetylation of aminoacyl-tRNAs. **a** Cell-free expression assays leading to the production of the control protein DHFR (red asterisk) from template DNA without toxins or with purified TacT, TacT2_STm_, TacT2_SEn_ or TacT3, added from the onset of the assay. [^14^C]Ac-CoA was added to all the samples. All samples were analysed by SDS-PAGE, and the production of DHFR was revealed by Coomassie staining (CM). tRNAs extracted from the samples were analysed by acid-urea PAGE and revealed by methylene blue staining (MB) and acetylation tracked by autoradiography (AR). Equivalent amount of toxins added to the sample was tested by western blotting against His tag of the toxins (WB). Quantification of inhibition of the production of DHFR (spotted bars) and acetylation of tRNAs (dashed bars) in all conditions is reported in the bar charts, where data represent the mean ± SEM (*n* ≥ 3). **b** Surface of the Ac-CoA-binding site of TacT3 with Ac-CoA represented as solid sticks for the orientation in TacT3 and shadowed for the orientation of Ac-CoA in TacT. Trp142 is in yellow and Gly145 is in red. **c** Exposure of tRNA molecules acetylated by TacTs to Pth treatment in vitro. tRNA molecules acetylated by different TacTs in vitro were subsequently incubated with purified Pth, acetylation was assessed by autoradiography, before or after samples were treated with purified Pth. All the samples were supplemented with [^14^C]Ac-CoA. Treated tRNA molecules were separated on acid-urea polyacrylamide gel and revealed by methylene blue staining (MB) (top panel). Acetylation was tracked by autoradiography (AR) (lower panel). **d** Model of activity of Tac toxins

We did not detect any noticeable effect of overexpression of *tacT2*_*STm*_ on *Salmonella* growth (Fig. 2a and Supplementary Fig. 4), however, since we observed a mild effect on persister formation (Fig. 1c) and a defect in recovery of macrophage-induced persisters in a single deletion mutant for *tacT2* (referred to as *ta9* in ref. ^11^), we considered that TacT2_STm_ was, nevertheless, a functional acetyltransferase. Each toxin, TacT2_STm_, TacT2_SEn_ and TacT3, was purified by co-expression with their cognate antitoxins to prevent inhibition of the growth of the recombinant *E. coli* strain. The toxins formed stable complexes with their antitoxins, that were later dissociated through denaturation on a nickel column, followed by toxin refolding. We investigated the effect of purified TacT2_STm_, TacT2_SEn_ and TacT3 in a cell-free expression assay using production of dihydrofolate reductase (DHFR) as a read-out of successful translation. All toxins inhibited production of DHFR when supplemented with Ac-CoA, although to different extents (Fig. 3a).

TacT acetylates the primary amine group of the amino acid on charged tRNA molecules, thereby blocking peptide bond formation and protein synthesis^17^. Similarly, purified TacT2_SEn_, TacT2_STm_ and TacT3 supplemented with [^14^C]Ac-CoA acetylated tRNA molecules in the cell-free expression samples (Fig. 3a) or extracted from *ΔtacATtacAT2tacAT3* deletion *Salmonella* (Fig. 3c), as revealed by the autoradiography after acid-urea PAGE. Remarkably, the extent of acetylation of tRNA molecules in the samples mirrored the extent of inhibition of DHFR production (Fig. 3a, bottom panels) and that of toxicity (Fig. 2a and data not shown). TacT3 appeared to be the most potent toxin, and comparison of its active site with that of TacT revealed a difference in the orientation of Ac-CoA (Fig. 3b). The adenine moiety is bent and more engaged in the binding pocket of TacT (and the modelled TacT2) compared to that of TacT3, most likely owing to the bulky side chain of the Trp142 residue present in TacT3. In TacT3, the adenine base of Ac-CoA is further stabilized outside the binding pocket by a hydrogen bond between its amine group and a carbonyl oxygen of the Gly145 residue (Fig. 3b). It is possible that this different conformation allows rapid unloading of coenzyme A after transfer of the acetyl moiety to the target substrate, increasing the processivity of the enzyme.

We previously showed that Peptidyl-tRNA hydrolase (Pth), which is an essential esterase of all bacterial species that recycles free peptidyl-tRNA molecules released during premature termination of translation^19^, detoxified the acetylated charged tRNAs^17^. Therefore, we tested whether the acetylation of TacT2_SEn_, TacT2_STm_ and TacT3-corrupted tRNAs could be resolved by Pth. When tRNA molecules acetylated by the toxins in vitro were subsequently incubated with purified Pth, a strong decrease in the radioactive acetylation signal was detected (Fig. 3c). In agreement, overexpression of *S*. Typhimurium *pth*, although having a detrimental effect on the bacterial growth, counteracted the effects of TacT2_SEn_ and TacT3 on growth inhibitory or persister induction (Supplementary Fig. 8).

Altogether, these results show that all TacT-like toxins present in *Salmonella* Typhimurium and Enteritidis block the primary amine group of the amino acid on charged tRNA molecules (Fig. 3d), and that they do this to different extent.

### TacT2_SEn_ acetylates aminoacyl-tRNAs more than TacT2_STm_

**Fig. 4:**
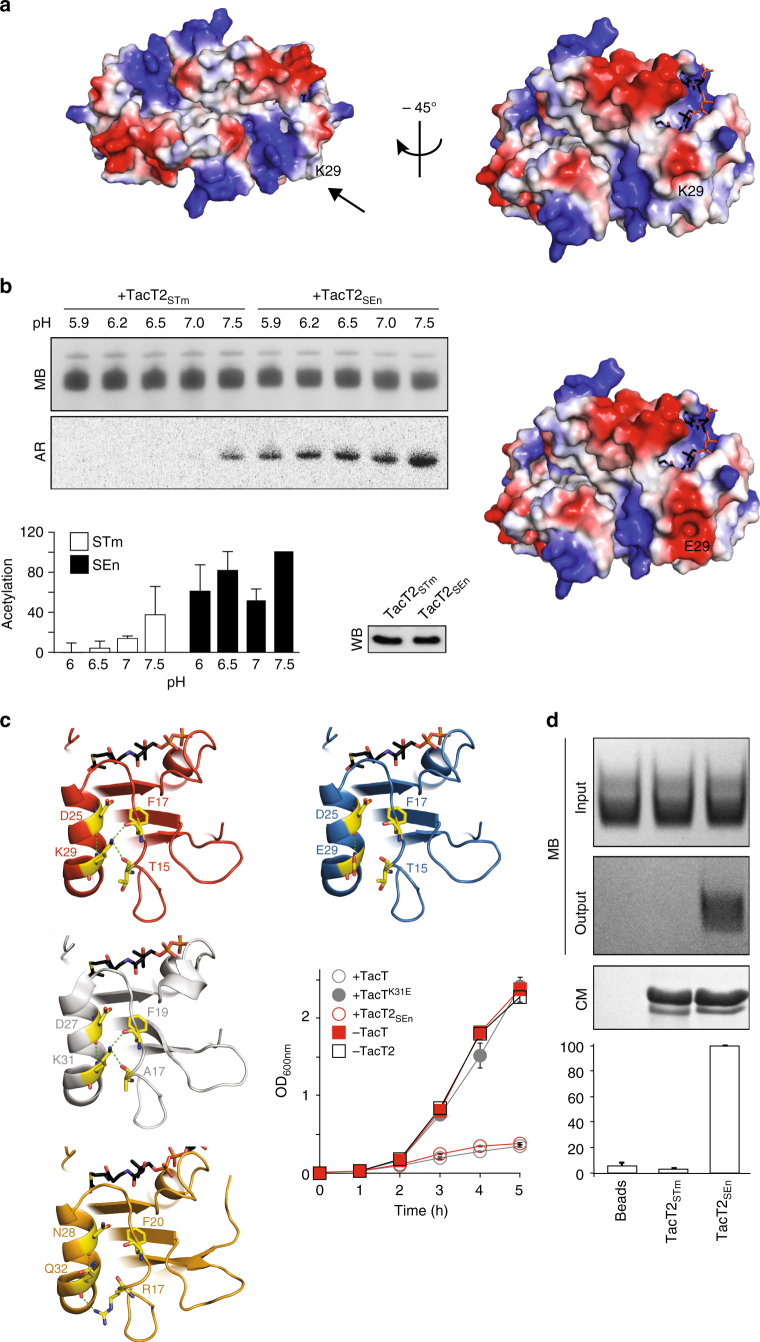
TacT2_SEn_ acetylates aminoacyl-tRNAs more efficiently than TacT2_STm_. **a** TacT2 polymorphism. Electrostatic potential of the surface of the modelled TacT2_SEn_ (top panels) or TacT2_STm_ (lower panel) dimer showing positive potential in blue and negative in red. Arrow points toward the polymorphic amino acid. A 45° rotation about the *y*-axis from the first view of TacT2_SEn_ showing the negative electrostatic potential of E29 in TacT2_STm_. **b** Extracted tRNA molecules from *S*. Typhimurium 12023 Δ*tacATtacAT2tacAT3* were treated with equivalent amounts of TacT2_STm_ or TacT2_SEn_ as assessed by western blotting. [^14^C]Ac-CoA was added to all samples from the onset of the assay and the pH of the reactions was set over a 5.9–7.5 range. Samples were analysed by acid-urea PAGE, tRNAs revealed by methylene blue staining (MB) (top panel) and acetylation tracked by autoradiography (AR) (lower panel). Quantification of acetylation of tRNAs in all conditions is reported in the bar chart, where data represent the mean ± SEM (*n* = 3). **c** TacT2_SEn_ (red) K29 and TacT (grey) K31 form two hydrogen bonds between the epsilon amine group of the lysine and carbonyl oxygens from peptide bonds on a neighbouring chain. In a model of TacT2_STm_ (blue), no hydrogen bond can be formed with E29. In TacT3 (orange), a stabilizing hydrogen bond is formed between Q32 and the side chain of the neighbouring R17. Growth curves of *S*. Typhimurium 12023 Δ*tacAT* expressing from pBAD33, *tacT* (+TacT), point mutant toxin (+TacT^K31E^) or carrying the empty vector (−TacT), or Δ*tacAT2* expressing from pBAD33 the Enteritidis wild-type toxin (+TacT2_SEn_) or carrying the empty vector (−TacT2). All cultures were supplemented with arabinose in fresh rich medium during lag phase. Data represent the mean ± SEM (*n* ≥ 3). **d** Binding of the two isoforms of TacT2 to tRNA molecules. Equivalent amounts of purified His-tagged inactive toxins TacT2_STm_^Y137F^ and TacT2_SEn_^Y137F^ were incubated with tRNA molecules extracted from *S*. Typhimurium 12023 Δ*tacATtacAT2tacAT3* and subsequently pulled down with anti-His antibody. The input and output tRNA molecules were analysed by acid-urea PAGE and revealed by methylene blue staining (MB—top panels). Equivalent pulldown of the two toxin isoforms was tested by SDS-PAGE and Coomassie staining (CM—lower panel). Quantification of the amount of tRNA molecules pulled down by the toxins is reported in the bar chart where data represent the mean ± SEM (*n* ≥ 3)

The activity of TacTs relies on several positively charged residues exposed at the surface of the molecule. Interestingly, TacT2 variability between serovars is limited to one amino acid with reversed charge (E29K), from negative in TacT2_STm_ to positive in TacT2_SEn_ at the surface of the molecule, in close proximity to the positive groove, leading to the active site (Fig. 4a). We investigated how this single amino acid change has an impact on the toxicity of TacT2.

Since our model proposed that the binding between the toxin and the target aminoacyl-tRNA(s) involves electrostatic bonds and the predicted pI of the two enzymes differ significantly (7.13 and 6.62 for TacT2_SEn_ and TacT2_STm_, respectively), we hypothesized that TacT2_STm_ could work better in acidic than neutral pH. We compared the enzymatic activities of TacT2_SEn_ and TacT2_STm_ over a pH range from 6.0 to 7.5. Aminoacyl-tRNAs extracted from Δ*tacATtacAT2tacAT3 Salmonella* and treated with purified TacT2_SEn_ supplemented with [^14^C]Ac-CoA were acetylated at all pH conditions tested. However, TacT2_STm_ only acetylated the aminoacyl-tRNAs efficiently at a pH of 7.5 (Fig. 4b). This result does not support a model of increased activity at acidic pH, but shows that TacT2_STm_ is more susceptible to pH changes than TacT2_SEn_.

Measurement of the thermostability of the two purified enzymes indicated a lowered Tm by at least 4 °C for TacT2_STm_, compared to TacT2_SEn_ (Supplementary Fig. 9), suggesting that TacT2_STm_ is a less stably folded protein. The model of the structure of TacT2_SEn_ suggests the presence of stabilizing hydrogen bonds formed between the side chain of K29 and the two neighbouring residues Phe17 and Tyr15 that cannot be formed by E29 in TacT2_STm_ (Fig. 4c red and blue structures, respectively). Interestingly, the structure of TacT^17^ reveals similar stabilizing bonds (K31 with Phe19 and Ala17—Fig. 4c, grey structure). Accordingly, a K31E substitution abolished toxicity of TacT in *Salmonella* (Fig. 4c, right panel), highlighting the importance of this region in the function of both of these enzymes. TacT3 does not contain a lysine in the corresponding region, but a Gln32 that engages in stabilizing hydrogen bonds with the side chain of the neighbouring Arg17 (Fig. 4c, orange structure). We hypothesized that in the absence of these stabilizing hydrogen bonds, the positively charged groove, leading to the active site of the toxin might be less accessible to the substrate aa-tRNA. We therefore used His-tagged, catalytically inactive TacT2 isoforms as potential substrate-trapping mutants, and tested the binding of TacT2_STm_^Y137F^ and TacT2_SEn_^Y137F^ to *Salmonella* aa-tRNA at pH 7.0. TacT2_SEn_^Y137F^ pulled down significantly more aa-tRNA than TacT2_STm_^Y137F^ (Fig. 4d), thereby reflecting the absence of activity of the Typhimurium isoform at pH 7.0 (Fig. 4b) and explaining the apparent higher toxicity of TacT2_SEn_ compared to TacT2_STm_.

### The Tac toxins target partially distinct subsets of aa-tRNAs

**Fig. 5:**
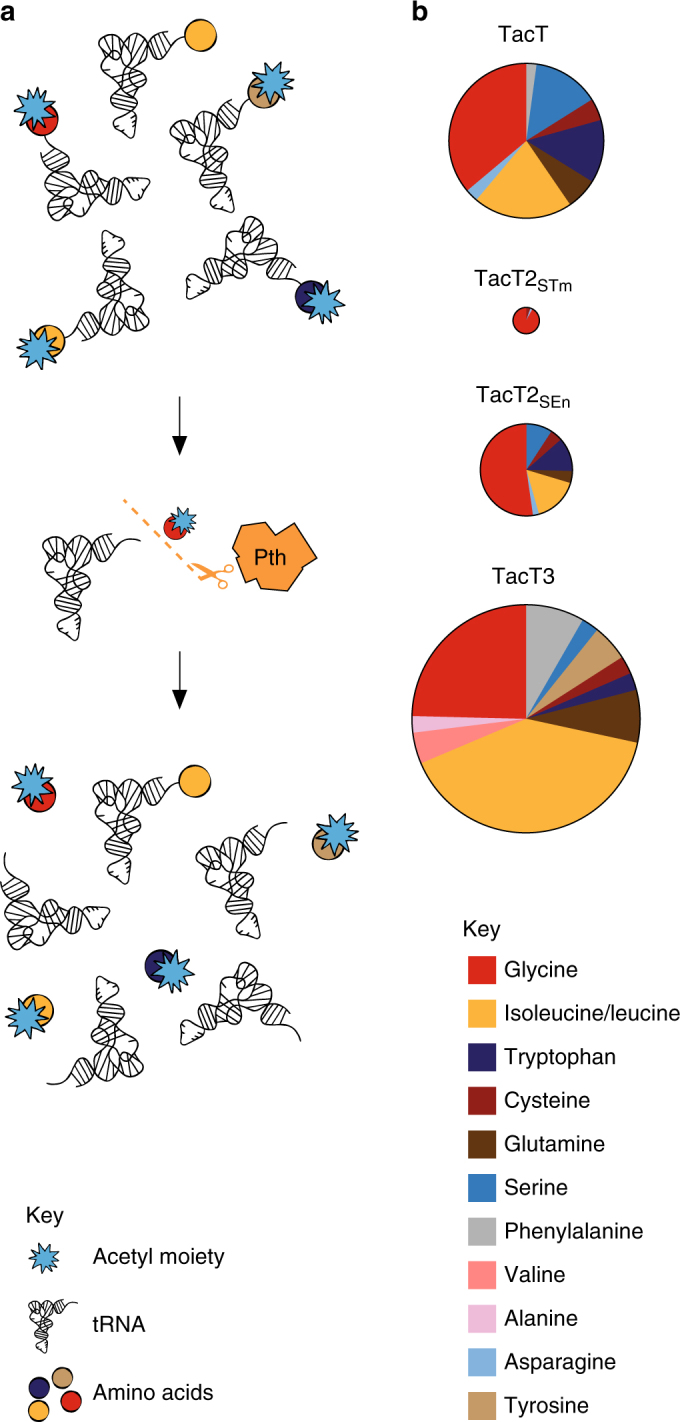
The three Tac toxins promote acetylation of specific subsets of aa-tRNAs identification of amino acids acetylated by the different toxins. **a** Toxin-corrupted aminoacyl-tRNAs were extracted from cell-free expression assays, and treated with purified Pth to release the acetylated amino acids from tRNAs and subsequently analysed by LC-MS. **b** Specificity of TacT, TacT2_SEn_, TacT2_STm_ and TacT3. Size of pie represents the relative amounts of acetylated amino acids recovered

We next investigated the specificity of activity of the three TacTs towards aa-tRNAs to understand better how they could work nonredundantly in persister formation. We added purified TacT, TacT2_SEn_, TacT2_STm_ or TacT3 toxin supplemented with Ac-CoA to cell-free expression assays and extracted the tRNA molecules from the samples to obtain acetylated aa-tRNAs among all RNA molecules. Then, relying on the ability of Pth to hydrolyze the ester bond between the acetylated amino acids and the tRNAs, we incubated the samples with purified Pth to liberate the corrupted amino acids. The amino acids were then detected by LC-MS (Fig. 5). The results indicated that following exposure to TacT, 13 different species of amino acids were detected as acetylated (Supplementary Fig. 10) with a strong bias towards Gly and Ile/Leu for all four toxins (Fig. 5 and Supplementary Fig. 10). We recovered different amounts of acetylated species for the four toxins (Fig. 5) that matched the extent of their acetylation (Fig. 3a). We investigated whether usage of specific aa-tRNAs by the translation machinery to synthesize DHFR in the cell-free translation assays introduced a bias in the acetylation preference of the toxins. Hence, we repeated the assay with or without the addition of the DHFR template in the samples. For each toxin, the repertoire of the acetylated species was identical in the presence or absence of active translation (Supplementary Figs. 10–11 and Supplementary Data 1), suggesting that usage of aa-tRNAs by translation machinery does not favour the activity of the toxins.

Altogether, these results show that the toxins can acetylate several, but not all amino acids, and even though the spectrum of acetylated species overlapped, the toxins differed in their substrate specificity.

## Discussion

This study extends our understanding of toxin-based growth arrest leading to persister formation of *Salmonella*. First, using clinical isolates, we show that *Salmonella* responsible for recurrent non-typhoidal invasive disease respond to internalization by primary human macrophages by dramatically increasing the proportion of persisters present in the population (Fig. 1a). The presence of persisters has been thought to contribute to the recalcitrance of many bacterial infections to antibiotic treatments. However, unambiguous evidence has been brought only recently by the study of invasive *Salmonella* infections by high-resolution single-nucleotide polymorphism (SNP) analysis. These studies led to differentiating reinfections (22%) from clonal relapses (78%) in the recurrence of infections (20–43%)^10^. The absence of accumulation of any SNP in the relapsing clones provided one of the strongest indications for the involvement of persisters in survival of antibiotic treatment and recurrence of the disease^10^. Our experiments here reveal that the formation of antibiotic-tolerant persisters during infection of macrophages is a common feature of several clinical *Salmonella* strains and confirm that *Salmonella* have evolved to respond to phagocytosis by promoting bacterial growth arrest with an approximately 1000-fold increase. This non-growing population, formed as part of a stress response from the bacteria confronted with host immune defence cells could constitute a reservoir for relapse of infection, since we previously showed that at least some of these growth-arrested bacteria can later resume growth, for example, upon death of the host cell.

Chromosomal class II TA modules were shown in *S*. Typhimurium 12023 to contribute to persister formation, and deletions of each of the single modules led to a defect in persister recovery after infection of macrophages^11^. Therefore, it was surprising to observe that *S*. Enteritidis strains, despite all lacking *relBE1* and *parDE* (Fig. 1b), form high levels of persisters in response to internalization by macrophages. We demonstrate that the single amino acid change, E29K, increases the activity of TacT2 towards promoting persister formation. The enhanced potency of TacT2_SEn_ might compensate for the loss of ParE and RelE1 and account for the ability of *S*. Enteritidis isolates to form persisters at a rate similar to that of *S*. Typhimurium.

TacT2 and TacT3 represent new acetyltransferase toxins^17, 20^ that acetylate the primary amine group of aminoacyl-tRNAs. It appears that the mechanism we first described with TacT^17^ to block translation is shared by other acetyltransferase toxins and might therefore represent a more general mechanism of growth control^20^.

It was surprising that TacT2_STm_ was not toxic upon overexpression in *Salmonella* (Fig. 2a). Interestingly, the same enzyme has been reported to be toxic under different overexpression conditions^21^. We show here that TacT2_STm_ has not lost the activity, since the purified toxin was shown to partially block cell-free translation and has a functional acetyltransferase activity (Fig. 3a). It is possible that this moderate toxicity might be revealed by much stronger overexpression^21^. This would be in agreement with our encountered difficulties in cloning *tacT2*_*STm*_ in high-copy strong expression vectors^22^ without accumulating mutations in the toxin gene.

This work also revealed the specificity of the three different toxins. The other well-characterized acetyltransferase toxin is AtaT^20^, which was shown to be extremely specific in acetylating only initiator ^Met^tRNA. Contrary to this, the Tac toxins target several elongator tRNAs and although there is a strong overlap, they have different target specificities, potentially explaining their non-redundancy. It will be interesting to investigate whether the three TacTs present in each strain of *Salmonella* are all active at the same time in the exact same cells, and if endogenous factors might influence their spectrum of targets in the bacterial cells, further contributing to a lack of redundancy.

## Methods

### Bacterial strains and media

The *S*. Typhimurium strains used in this study were wild-type 12023s and its mutant derivatives and clinical strains (Supplementary Tables 1 and 4). The *S*. Enteritidis strains used in this study are listed in Supplementary Tables 1 and 4. The *E. coli* expression strain was BL21 (Invitrogen). All strains were grown at 37 **°**C in fully aerated rich growth medium (Luria Bertani) or M9 minimal medium, supplemented when appropriate with 100 μg/ml ampicillin, 25 μg/ml chloramphenicol, 50 μg/ml kanamycin, 0.2% L-arabinose and/or 0.5–1 mM IPTG to allow production of recombinant proteins.

### Preparation of primary macrophages

Whole-blood samples were taken from human donors with informed consent and were registered, stored and disposed of according to Imperial College Healthcare Tissue Bank Policy. The authority granted by the Research Ethics Committee to the Tissue Bank to hold a Human Tissue licence and approve work at Imperial using human tissue ensures that the use of human samples is covered by college-wide ethical approval and the Human Tissue Act, 2004. Samples were taken using aseptic technique into sodium heparin BD vacutainers. Blood samples were centrifuged at 550 × *g* for 45 min at room temperature. The top plasma layer was removed and the blood pellet was resuspended in PBS before layering slowly on 20 ml pre-settled Histopaque-1077. The layered samples were then centrifuged at 450 × *g* for 45 min at RT. The top layer was removed and the peripheral blood mononuclear cells (PBMC) layer harvested using a Pasteur pipette. The PBMCs were washed three times with 45 ml of pre-warmed RPMI and the resulting red blood cell pellet was lysed at 37 **°**C in 10 ml lysis buffer (1.5 M NH_4_Cl, 0.1 M NaHCO_3,_ 0.01 M disodium EDTA 0.01 M). The remaining cells were pelleted and resuspended in a suitable volume of pre-warmed RPMI. Primary monocytes were isolated from PBMCs, working swiftly, on ice and using pre-cooled solutions. Cells were passed through a 30 μM nylon mesh, and 20 μl of CD14 beads (MACS, Miltenyi Biotec) were added to the samples of 10^7^ cells in 80 μl of degassed buffer (PBS, 0.5% BSA, 2 mM EDTA) and incubated for 15 min. Cells were washed in 2 ml buffer and centrifuged at 300 × *g* for 10 min. The supernatant was aspirated and the pellet was resuspended in 50 μl buffer. CD14+ monocytes were enriched over an MACS column, as per the manufacturer’s instructions. Monocytes were resuspended in RPMI-1640 (30 ml per 3–5 × 10^7^ cells) with 5 ml L-glutamate and 10% autologous human serum. Autologous serum was prepared by allowing the whole-blood samples to clot at room temperature for 45 min, followed by centrifugation and collection of the serum.

### Rates of protein synthesis

Cells were grown at 37 °C in LB to stationary phase overnight. Cultures were then diluted to OD_600_ of 0.1 into fresh LB medium supplemented with 10 mM IPTG or chloramphenicol (30 µg/ml to inhibit protein synthesis) and were incubated at 37 °C with aeration. At 5, 30 and 60 min, 500 µl samples were harvested, normalized to an OD_600_ of 0.1 and incubated at room temperature, with 1 µCi of methionine-^35^S. After 5 min of incorporation of radio-labelled isotope, the samples were chased for 10 min with 0.5 mg of the cold isotope. Cells were then washed with 500 µl 70% ethanol three times. Pellets were then resuspended in 10 µl 70% ethanol, dotted onto Whattman paper and exposed to a photostimulable phosphor (PSP) plate overnight. Amounts of incorporated radioactivity were then visualized using a phosphorimager and quantified with ImageJ.

### Expression and purification of recombinant TacT2^Y137F^ toxins

*Salmonella* TacT2_SEn_^Y137F^ and TacT2_STm_^Y137F^ sequences encoding the residues were expressed as a N-terminal (His)7 fusion protein of the pQlinkH::TacT2^Y137F^ vector. *E. coli*, strain PC2^23^, was grown in LB at 37 °C until an OD_600_ of 0.8 was reached, then protein expression was induced overnight at 18 °C by addition of 0.5 mM IPTG. Cells were lysed by sonication in the lysis buffer (50 mM Tris pH 7.5, 500 mM NaCl, 1 mM phenylmethanesulfonyl fluoride (PMSF)). Cleared lysate was incubated with Ni-NTA resin (Thermo Fisher) for 2 h at 4 °C with agitation. The lysate/resin mixture was applied to a column and washed with wash buffer (50 mM Tris pH 7.5, 500 mM NaCl, 20 mM imidazole) and the protein was eluted in 10 ml elution buffer (50 mM Tris pH 7.5, 500 mM NaCl, 500 mM imidazole) and subjected to dialysis (50 mM Tris pH 7.5, 500 mM NaCl).

### Analysis of tRNA by gel electrophoresis

Total RNA was extracted from *Salmonella* under acidic conditions to maintain the ester link between tRNA and amino acid/peptide, as described in ref. ^24^. This allows isolation of aminoacyl-tRNAs molecules^25^. aa-tRNA were separated by acid-urea PAGE, as described in ref. ^25^ and stained by methylene blue (500 mM sodium acetate, 0.06% methylene blue).

### Pth purification and in vitro functional assay

Pth was purified as reported in ref. ^17^. The activity of Pth was assessed, as previously reported in ref. ^26^. Briefly, purified modified tRNAs were incubated with purified Pth (4 μg/ml) in activity assay buffer (10 mM Tris acetate, 10 mM magnesium acetate, 20 mM ammonium acetate pH 8.0) 1 h at 37 °C.

### Expression and purification of recombinant functional toxins

*Salmonella* TacT, TacT2_SEn_, TacT2_STm_ or TacT3 (with an N-terminal (His)6 purification tag) and cognate antitoxin sequence encoding residues were expressed from the dual expression vector pRSFduet. *E. coli* PC2 strain was grown in LB at 37 °C until an OD_600_ of 0.8 was reached, then protein expression was induced overnight at 18 °C by addition of 0.5 mM IPTG. Cells were lysed by sonication in lysis buffer (50 mM Tris pH 7.5, 500 mM NaCl, 0.5 mM PMSF). Cleared lysate was incubated with NiNTA resin (Qiagen) for 1 h at 4 °C with agitation. The lysate/resin mixture was applied to a column and washed with wash buffer (50 mM Tris pH 7.5, 500 mM NaCl, 20 mM imidazole).

The TA complex bound to the column was denatured with 50 ml denaturation buffer (50 mM Tris pH 7.5, 500 mM NaCl, 5 M GnHCl) and then 50 ml of denaturation wash buffer (50 mM Tris pH 7.5, 500 mM NaCl, 5 M GnHCl, 20 mM Imidazole). Finally, the denatured toxin was eluted in 10 ml denaturation elution buffer (50 mM Tris pH 7.5, 500 mM NaCl, 5 M GnHCl, 500 mM Imidazole). The denatured protein was refolded via overnight dialysis at 4 °C in 2 l dialysis buffer (25 mM Tris pH 7.5, 25 mM NaCl, 5% glycerol) using 3500 MWCO dialysis tubing (Spectrum labs). Refolded TacT1–3 was concentrated and stored at −80 °C.

### Cell-free expression assay

In vitro cell-free expression assays were carried out using the PURExpress In Vitro Protein Synthesis Kit (New England Biolabs [NEB], Massachusetts, USA), following the manufacturer’s guidelines, with the exception of the addition of purified toxins (2 ng) supplemented or not with Ac-CoA (2 mM—Sigma-Aldrich) or ^14^C Ac-CoA (0.2 mM, 3.7 mBq—Perkin Elmer, Massachusetts, USA) where specified. Samples were analysed by SDS-PAGE or by acid/urea PAGE and visualized by Coomassie Blue staining or by autoradiography.

### Persister assays

Bacterial strains were grown to stationary phase in M9 minimal medium overnight, then diluted to an OD_600_ of 0.05 into fresh M9 minimal medium, supplemented with antibiotics as appropriate. 1 mM IPTG and 0.2% L-arabinose was used to induce expression of genes from pCA24N and pBAD33 vectors, respectively, and cultures were incubated at 37 °C. One hour after induction, samples were taken and CFU enumerated (*t* = 0 h). Cefotaxime (100 µg/ml) was added to the medium and cultures were incubated for 4 h at 37 °C. After the cefotaxime treatment, 1 ml samples were collected and the surviving bacteria enumerated after the antibiotic was washed out (*t* = 4 h).

Macrophage-induced persisters were recovered as follows: after 30 min infection of primary human macrophages with stationary phase bacteria grown overnight in LB^27^, macrophages were lysed and intracellular bacteria collected, washed once in PBS and resuspended in fresh LB containing various antibiotics. The fold increase in persisters caused by internalization in macrophages was quantified by calculating the ratio of percentage of survival of the macrophage-exposed population over that of the same population used for the macrophage inoculum, subsequently grown only in LB containing antibiotics (LB persisters).

### Crystallization and structure determination of TacT3^Y143F^

The purified TacT3^Y143F^ proteins (40 mg/ml) crystallized by sitting drop vapour diffusion against a reservoir of 10% PEG 6000, 100 mM Bicine NaOH pH 9.0. Crystals were harvested and submerged in a crystallization buffer containing 20% glycerol for 10 s followed by freezing by immersion into liquid nitrogen. Diffraction data were acquired at diamond light source (Oxfordshire, UK) on beamline I04–1. High-resolution native data sets were auto-integrated using autoPROC software^28^. Phases was obtained with PHENIX Phaser software using molecular replacement function and a polyalanine scaffold of TacT (PDB 5FVJ) as a model^29^. The model was then modified using AutoBuild^30^ and manually improved using Coot^31^ and refined using PHENIX and Refmac^32, 33^ against the high-resolution native data to give a final structure with an *R*_free_ of 23.1% and good geometry (Supplementary Table 2). The relatively high final *R* factor could be explained by the flexible loop in the 75–82 region in one of the monomer units.

### Pulldown assay

TacT2_SEn_^Y137F^ or TacT2_STm_^Y137F^ (200 µg) were immobilized on 25 µl of Ni-NTA resin (Pierce) followed by extensive washing with buffer A (100 mM Tris pH 8, 150 mM sodium chloride). The beads were then equilibrated in buffer B (100 mM HEPES pH 7.5, 150 mM potassium chloride, 5 mM magnesium sulphate, 10 mM ammonium acetate) and incubated with agitation overnight at 4 °C in the presence of 100 µg of total RNA purified from *Salmonella* Typhimurium 12023 Δ*tacATtacAT2tacAT3*. After extensive washing with buffer B, the beads were split 1:9. 10% of total beads was boiled with protein sample buffer followed by SDS-PAGE and Coomasie Blue staining, the remaining 90% was subjected to tRNA extraction under acidic conditions as described previously^24^. The resulting tRNA was separated by acid-urea PAGE as described in ref. ^25^ and stained by methylene blue (500 mM sodium acetate, 0.06% methylene blue).

### Differential scanning fluorescence (DSF) assay

TacT2_SEn_^Y137F^ or TacT2_STm_^Y137F^ variants were diluted in 96-well plates to a concentration of 10 µM in a range of buffers (100 mM of MES pH 5.0, MES pH 6.0, HEPES pH 7.0, sodium phosphate pH 7.4, sodium phosphate pH 8.0, or CHES pH 9.0) all containing 500 mM sodium chloride and 10 × SYPRO Orange dye (Sigma) in a total volume of 30 µl. A step-wise thermal ramp from 25 to 95 °C was obtained using Mx3005p instrument (Agilent) and melting temperatures (Tm) were calculated according to established protocols^34^.

### Detection of acetylated amino acids

Total RNA was extracted from the cell-free expression assay under acidic conditions to maintain the ester link between tRNA and amino acid/peptide as described in ref. ^17^. Purified RNA (10 μl) was incubated with 25 µg purified Pth in buffer (10 mM Tris acetate, 10 mM magnesium acetate, 20 mM ammonium acetate pH 8.) for 1 h at 37 °C. Samples were combined 1:1:2 with solvent X (40:40:20 acetonitrile:methanol:water) and solvent Y (0.2% acetic acid in acetonitrile), vortexed and precipitate removed via centrifugation. Aqueous normal phase liquid chromatography was performed using an Agilent 1290 Infinity II LC system equipped with a binary pump, temperature-controlled auto-sampler (set at 4 °C) and temperature-controlled column compartment (set at 25 °C), containing a Cogent Diamond Hydride Type C silica column (150 mm × 2.1 mm; dead volume 315 µl). A flow-rate of 0.4 ml/min was used. Elution of polar metabolites was carried out using solvent A consisting of deionized water (resistivity ~18 MΩ cm) containing 0.2% acetic acid, and solvent B consisting of 0.2% acetic acid in acetonitrile. The following gradient was used: 0 min 85% B; 0–2 min 85% B; 3–5 min to 80% B; 6–7 min 75% B; 8–9 min 70% B; 10–11 min 50% B; 11.1–14 min 20% B; 14.1–25 min hold 20% B follow by a 5 min re-equilibration period at 85% B at a flow-rate of 0.4 ml/min. Accurate mass spectrometry was carried out using an Agilent Accurate Mass 6545 QTOF apparatus. Dynamic mass axis calibration was achieved by continuous infusion, post-chromatography, of a reference mass solution using an isocratic pump connected to an ESI ionization source, operated in the positive and negative-ion modes. This instrument enabled accurate mass spectral measurements with an error of less than 5 parts-per-million (ppm), mass resolution ranging from 10,000 to 25,000 over the *m*/*z* range of 118–955 atomic mass units, and a 100,000-fold dynamic range with picomolar sensitivity. The data were collected in the centroid 4 GHz (extended dynamic range) mode. Detected *m*/*z* were deemed to be identified metabolites on the basis of unique accurate mass-retention time identifiers for masses exhibiting the expected distribution of accompanying isotopomers (Supplementary Table 3). Typical variation in abundance for most of the metabolites stayed between 5 and 10% under these experimental conditions. Targeted metabolomics was performed using Agilent Mass Hunter Qualitative Analysis B.07.00.

### Data availability

The coordinates for the structural model of TacT3^Y143F^ have been deposited in the Protein Data Bank under ID code PDB: 6G96.

## Electronic supplementary material

Supplementary Information

Description of Additional Supplementary Files

Supplementary Data 1
