# Supplementary material for: Activity of acetyltransferase toxins involved in Salmonella persister formation during macrophage infection
Source: Nat Commun. 2018 May 18;9:1993. doi: 10.1038/s41467-018-04472-6 (PMC5959882; doi:10.1038/s41467-018-04472-6)
Supplement: Supplementary file 1 — Supplementary Information [file 41467_2018_4472_MOESM1_ESM.pdf]

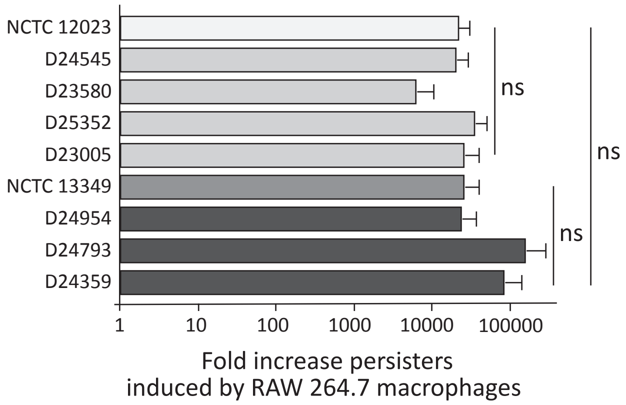

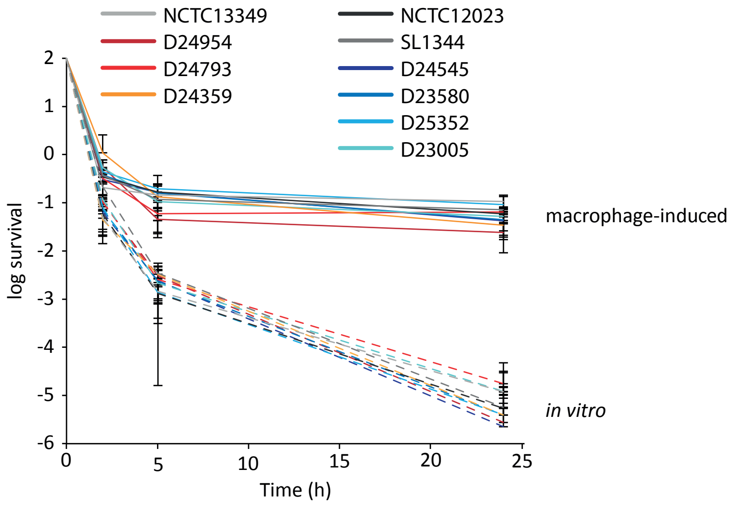

Supplementary Figure 1 - Top panel - Fold increase in persisters caused by 30 min internalization in RAW 264.7 macrophages relative to levels in inocula, measured after exposure to gentamicin. Lower panel represents killing kinetics with (macrophage-induced) or without (in vitro) 30 min internalization in RAW 264.7 cells. Data represent the mean ± SEM (n ≥3) and were analysed using one-way ANOVA (ns, non-significant).

```
                                          *
(T6)TacT3  MMFTDWHEAAIGKTHNRMNFDCGDADLNQFLQRHARQNHEKGTTKTYVALDNSDVTRIHGFYSVSPASLIYAQVPGAISKGLGRYDVPVF 89
(T8)TacT   -MGRVTAPEPLSAFHQVAEFVSGEAVLDDWLKQKGLKNQALGAARTFVVCK-KDTKQVAGFYSLATGSVNHTEATGNLRR-NMPDPIPVI 87
(T9)TacT2  ---MISTPEPLHAGHILTPFCCGVDSIDNWLEQRAMKNQTTGASRTFVCCG-SDS-NVLAYYSLASSAVTTNTSPGRFRR-NMPDPIPVV 84
                                          K

(T6)TacT3  RLGRLAVDKSMQGQGLGAQLLLSAGKRCIQAALQVGGVALLIDAKNKQVCDWYKGFGAVPLNDQPLSLLLSFKTLYAALSASGRL  173
(T8)TacT   ILARLAVDLSFHGKGLGADLLHDAVLRCYRVAENIGVRAIMVHALTEEAKNFYIHHGFKSSQTQQRTLFLRLPQ-----------  161
(T9)TacT2  VLGRLAVDKSLHGQGVARALVRDAGLRVIQVAETIGIRGMLVHALSDEAREFYQRVGFVPSPMDPMMLMVTLGDLVESV------  163
```

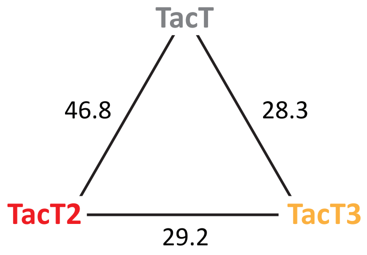

Supplementary Figure 2 - Top panel - Amino acid sequence alignment (Clustal Omega). Lower panel - identity matrix of three S. Typhimurium GNAT toxins, TacTs. Yellow highlights – Ac-CoA binding residues; orange highlights – residues known to be involved in TacT activity; grey highlight – catalytic tyrosine involved in transfer of acetyl moiety; blue letters – identical residues; red letters – similar residues; asterisk – TacT2 polymorphism.

A

	WT	-TacT2	+TacT2	-TacT3	+TacT3
Cefotaxime	5	5	5	5	5

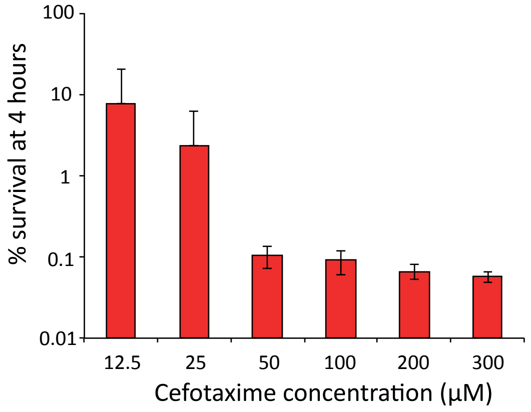

B

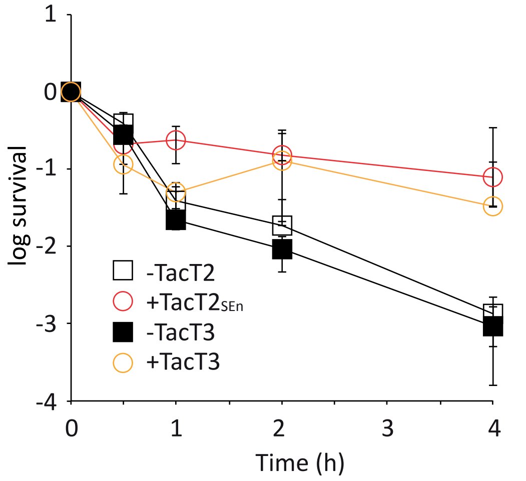

Supplementary Figure 3 - MIC of cefotaxime (µg/ml) (top panel) and rate of survival of S. Typhimurium 12023 ΔtacAT2 as a function of concentration of cefotaxime (lower panel) (A) and killing kinetics with bactericidal concentrations (100 µg/ml) of cefotaxime (B) in cultures of S. Typhimurium 12023 ΔtacAT2 (-TacT2), 12023 ΔtacAT2 carrying pBAD33::tacT2SEn (+TacT2SEn), 12023 ΔtacAT3 (-TacT3) or 12023 ΔtacAT3 carrying pBAD33::tacT3 (+TacT3). Arabinose was added to all cultures in fresh medium during lag phase and the antibiotic treatment started 1 h later. Data represent the mean ± SEM (n=3).

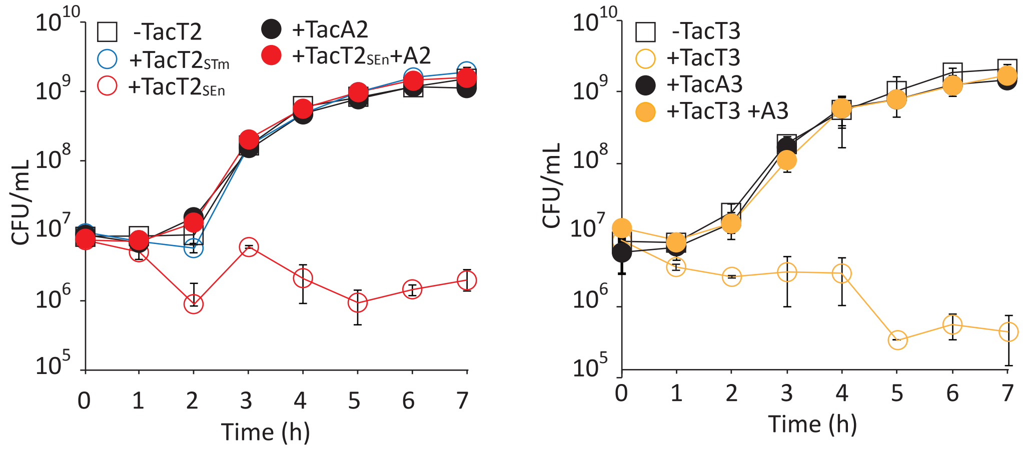

Supplementary Figure 4 - Left panel - Growth curves of S. Typhimurium 12023 ΔtacTA2 carrying pBAD33 (-TacT2), pBAD33::tacT2STm (+TacT2STm), pBAD33::tacT2SEn (+TacT2SEn), pCA24N::TacA2 (+TacA2) or pBAD33::tacT2SEn and pCA24N::tacA2 (+TacT2SEn+TacA2). Right panel - Growth curves of S. Typhimurium 12023 ΔtacTA3 carrying pBAD33 (-TacT3), pBAD33::tacT3 (+TacT3), pCA24N::TacA3 (+TacA3) or pBAD33::tacT3 and pCA24N::tacA3 (+TacT3+TacA3). All cultures were supplemented with arabinose and IPTG in fresh rich medium during lag phase and growth was monitored by CFU.

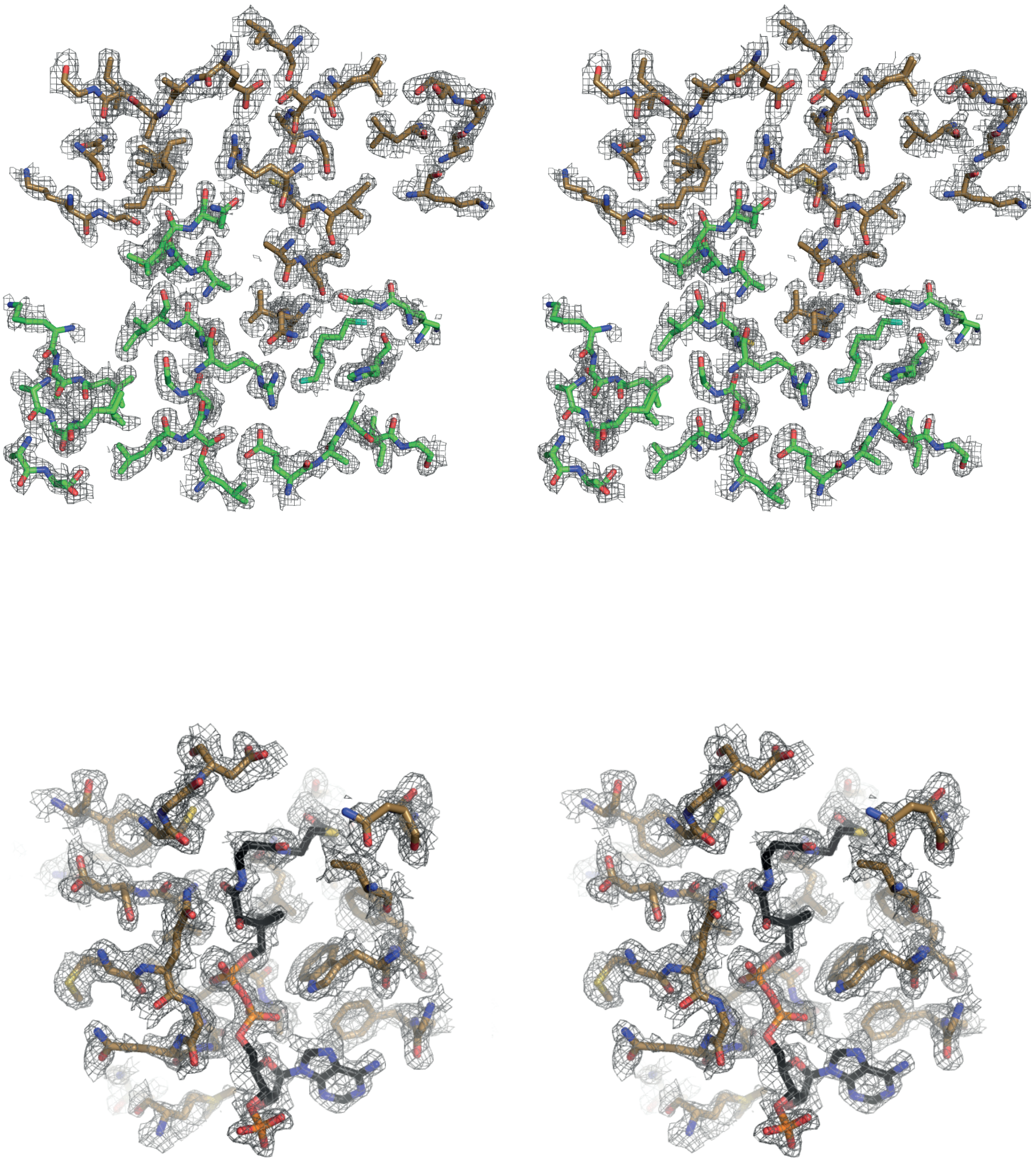

Density modification (2Fo-Fc) maps 1.0 σ

Supplementary Figure 5 - Final TacT3Y143F structure in the 2Fo-Fc electron density maps at 1.0 σ. Protein chains are colored brown and green and Ac-CoA in black.

A

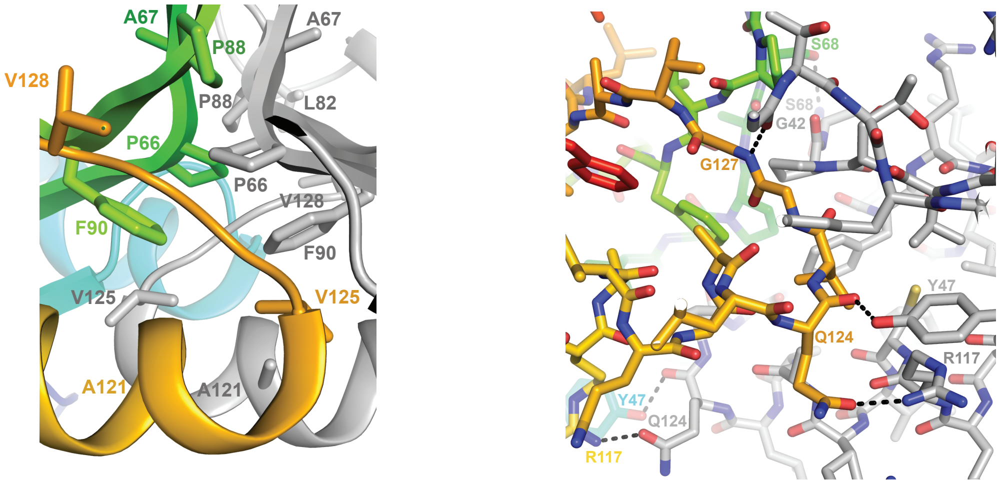

B

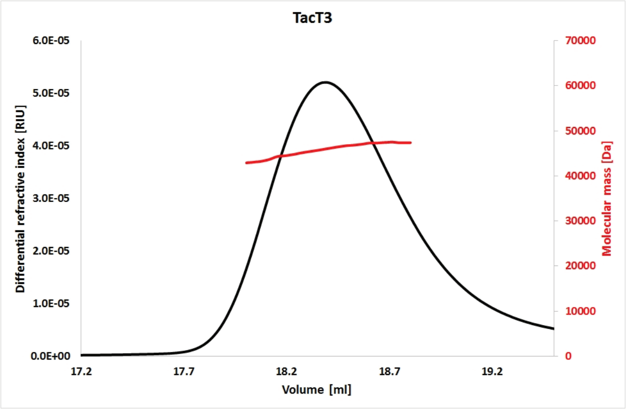

Supplementary Figure 6 - (A) Details of the dimer interface of TacT3. Colours and view are conserved from Figure 2C (chain A rainbow, chain B grey). The left panel highlights hydrophobic sidechains buried at the interface, whereas the right panel shows hydrogen bonds stabilizing the interface.
(B) SEC-MALLS showing the elution profile of TacT3 (black line) and predicted molecular weight (red line).

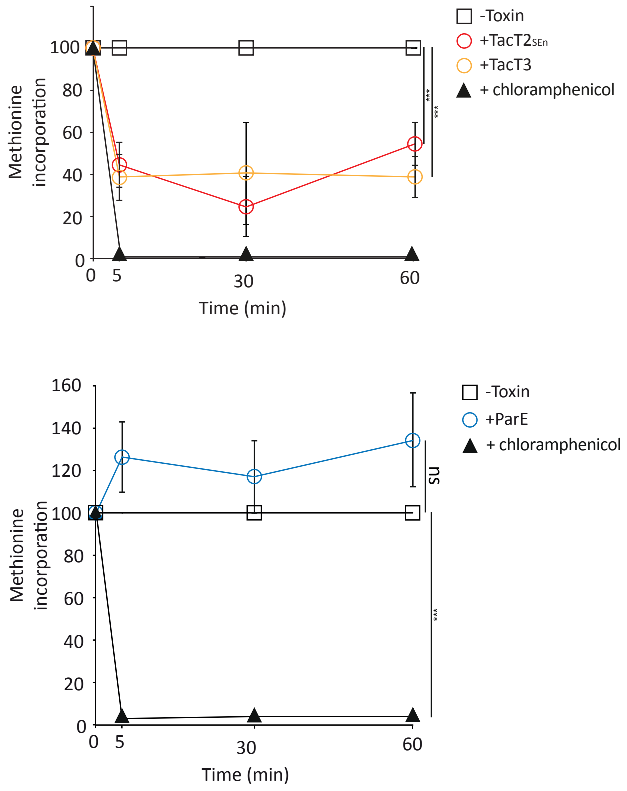

Supplementary Figure 7 - Levels of incorporation of radiolabeled methionine during pulse chase assays in lag phase cultures of (top panel) S. Typhimurium 12023 ΔtacTA2tacTA3 carrying pBAD33 (-Toxin), pBAD33::tacT2SEn (+TacT2SEn), pBAD33::tacT3 (+TacT3) or treated with bacteriostatic concentration of chloramphenicol or (lower panel) S. Typhimurium 12023 ΔparDE carrying pBAD33 (-Toxin), pBAD33::parE (+ParE) or treated with bacteriostatic concentration of chloramphenicol. All cultures were supplemented with arabinose at t0 in fresh minimal medium during lag phase. All measures were normalized to those of the negative control samples.
Data represent the mean ± SEM (n≥3) and were analysed using a Student's t test (***$p<0.005$).

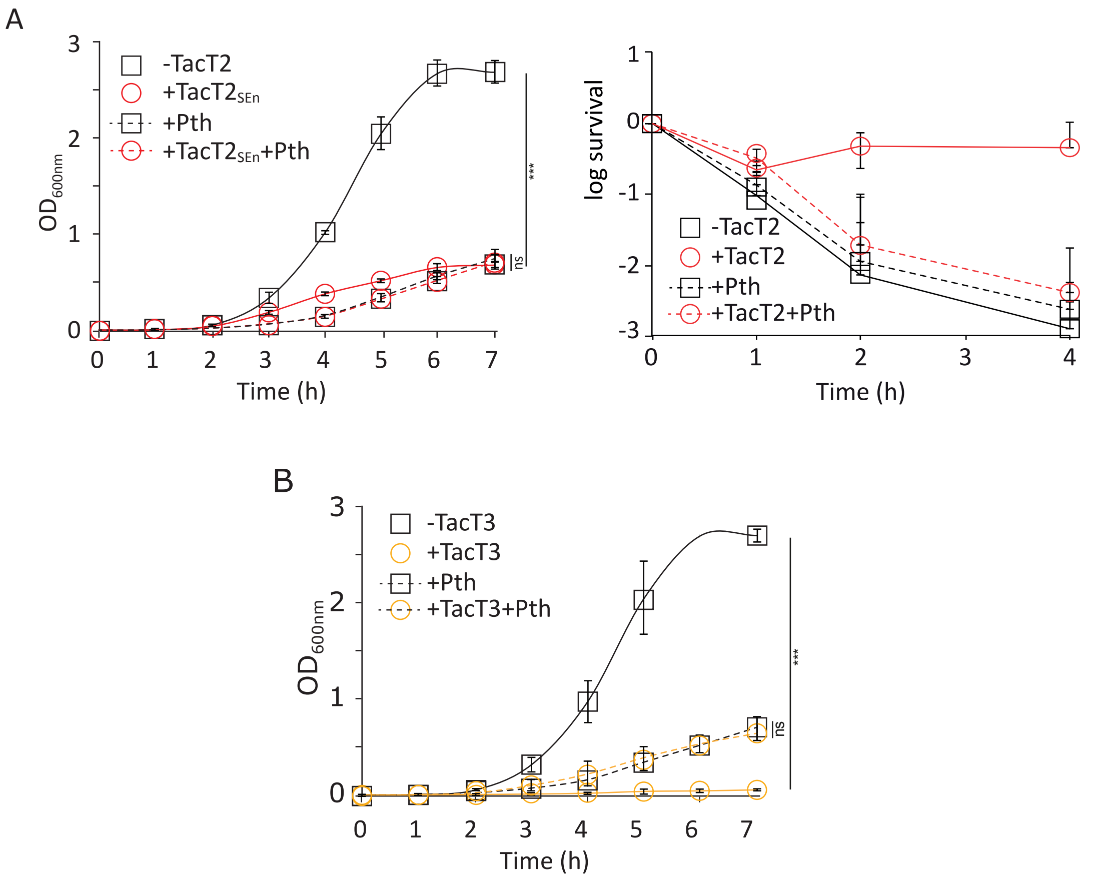

Supplementary Figure 8 - (A) Growth curves (left panel) or killing kinetics with bactericidal concentrations (100 μg/ml) of cefotaxime (right panel) of S. Typhimurium 12023 ΔtacAT2 (-TacT2), ΔtacAT2 carrying pBAD33::tacT2SEn (+TacT2SEn), pCA24N::pth (+Pth), or pBAD33::tacT2SEn and pCA24N::pth (+TacT2SEn+Pth). (B) Growth curves of S. Typhimurium 12023 ΔtacAT3 (-TacT3), ΔtacAT3 carrying pBAD33::tacT3 (+TacT3), pCA24N::pth (+Pth), or pBAD33::tacT3 and pCA24N::pth (+TacT3+Pth).

All cultures were supplemented with arabinose and IPTG in fresh rich medium during lag phase. Data represent the mean ± SEM (n =3) and were analysed using a Student's t test (ns, non-significant; ***p<0.005).

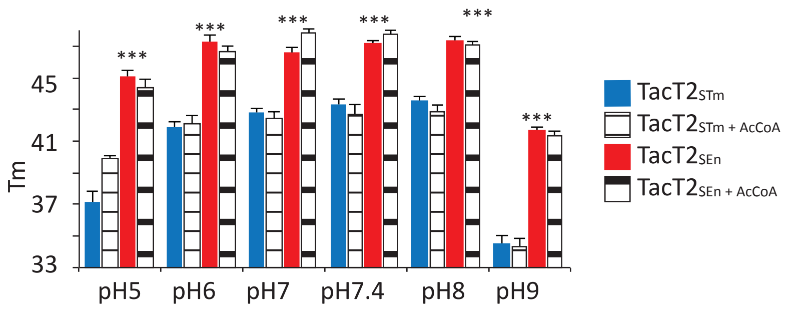

Supplementary Figure 9 - Measure of the thermostability of purified TacT2SEn and TacT2STm by differential scanning fluorescence in presence or absence of Ac-CoA and at different pH ranging 5-9.

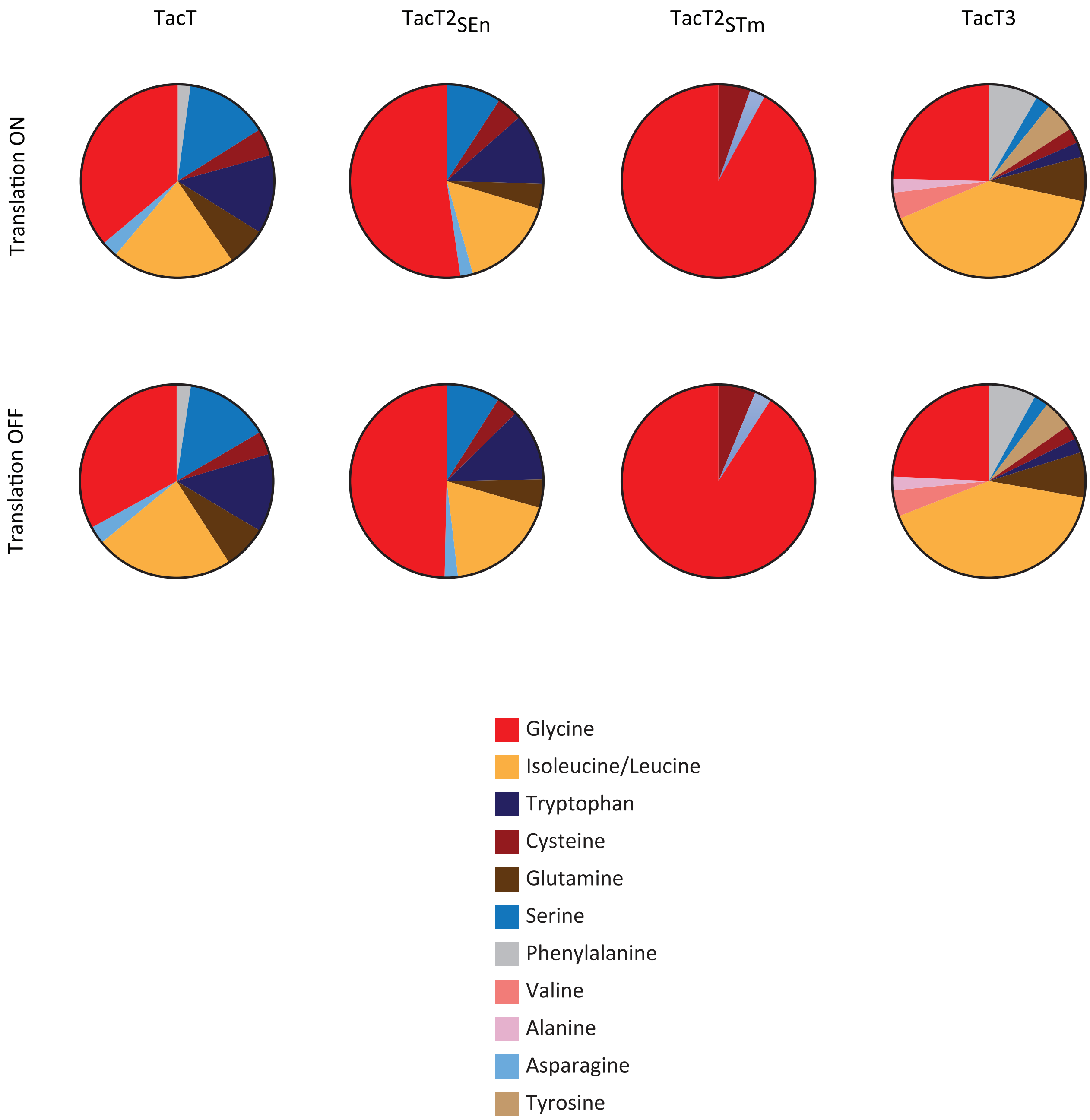

Supplementary Figure 10 - LC-MS identification of amino acids acetylated by TacT, TacT2SEn, TacT2STm or TacT3 and represented as proportion of all acetylated species.

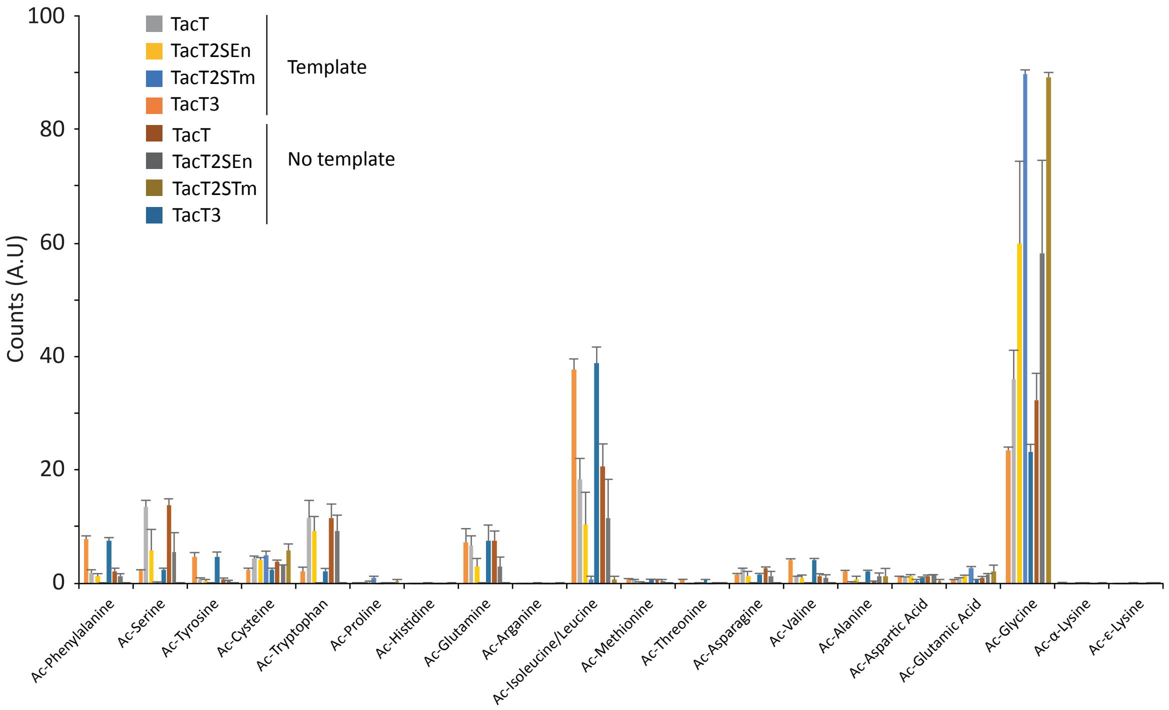

Supplementary Figure 11 - LC-MS identification of amino acids acetylated by TacT, TacT2SEn, TacT2STm or TacT3. Data represent the mean ± SEM (n =3).

		S. Typhimurium				*S.* Enteritidis		
**Isolate name**		**D24545**	**D23580**	**D25352**	**D23005**	**D24954**	**D24793**	**D24359**
Date of isolate		01-03-04	12-01-04	18-04-04	16-12-03	25-03-04	17-03-04	20-02-04
Age of patient (months)		18	26	40	12	7	12	12
Gender of patient		F	F	M	F	M	F	F
Malaria parasite count (%RBCs)		3	3	negative	negative	negative	negative	negative
Malnutrition		Y	N	N	N	N	N	N
ADCMK sensitivity		SS	S	RR	RR	SS	S	RR
Beta-lactams	Amoxicillin	R	R	R	R	R	S	S
	Coamoxiclav	S	S	S	S	S	S	S
	Cefuroxime	R	R	R	R	R	R	R
	Cefotaxime	S	S	S	S	S	S	S
	Ceftazidime	S	S	S	S	S	S	S
Aminoglycosides	Gentamycin	S	S	S	S	S	S	S
	Kanamycin	S	S	S	S	S	S	S
Other antibiotics	Chloramphenicol	R	R	R	R	R	S	S
	Cotrimoxazole	R	R	R	R	R	S	S
	Ciprofloxacin	S	S	S	S	S	S	S
	Tetracycline	S	S	S	S	R	S	S
	Rifampicin	R	R	R	R	R	R	R

Supplementary Table 1 - Clinical isolates used in the study

**Data Collection**	
Space group	P $2_1 2_1 2_1$
Cell dimensions: a, b, c [Å]	64.8, 71.2, 86.3
Cell dimensions: α, β, γ [˚]	90, 90, 90
Resolution [Å]	86.31-1.48 (1.48-1.5)
No. of unique reflections	67550 (6635)
R_{pim}	0.094 (0.901)
I/σI	8.7 (2.2)
Completeness[%]	99.77 (99.76)
Redundancy	6.6 (6.2)
**Refinement**	
Resolution [Å]	54.93-1.48
No. of reflections	67470
R_{work}/R_{free}	0.209 / 0.231
No. of protein atoms	2798
No. of ligand/ion atoms	102
No. of water atoms	454
B-factor: protein	20.62
B-factor: ligand/ion	17.76
B-factor: water	34.91
RMSD: bond lengths [Å]	0.0063
RMSD: angles [˚]	1.047
Ramachandran plot: favored [%]	99.42
Ramachandran plot: allowed [%]	0.58
Ramachandran plot: outliers [%]	0

Supplementary Table 2 - TacT3Y143F data collection and refinement statistics

**Compound**	**Chemical Formula**	**Theoretical m/z**	**Experimental m/z**	**Retention Time (minutes)**
N-Acetyl-Phenylalanine	$C_{11}H_{13}NO_3$	208.0968	208.0971	0.958-1.189
N-Acetyl-Leucine	$C_8H_{15}NO_3$	174.1125	174.1124	1.023-1.221
N-Acetyl-Serine	$C_5H_9NO_4$	148.0604	148.0611	1.318-1.566
N-Acetyl-Tyrosine	$C_{11}H_{13}NO_4$	224.0917	224.0908	1.017-1.199
N-Acetyl-Cysteine	$C_5H_9NO_3S$	164.0376	164.0375	1.018-1.233
N-Acetyl-Tryptophan	$C_{13}H_{14}N_2O_3$	247.1077	247.1081	0.992-1.157
N-Acetyl-Proline	$C_7H_{11}NO_3$	158.0812	158.0812	1.305-1.52
N-Acetyl-Histidine	$C_8H_{11}N_3O_3$	198.0873	198.0875	11.746-12.127
N-Acetyl-Glutamine	$C_7H_{12}N_2O_4$	189.087	189.0871	1.679-1.927
N-Acetyl-Arganine	$C_8H_{16}N_4O_3$	217.1295	217.1295	12.225-12.539
N-Acetyl-Methionine	$C_7H1_3NO_3S$	192.0689	192.0692	1.036-1.251
N-Acetyl-Threonine	$C_6H_{11}NO_4$	162.0761	162.076	1.267-1.499
N-Acetyl-Asparagine	$C_6H_{10}N_2O_4$	175.0713	175.0714	1.576-1.807
N-Acetyl-Valine	$C_7H_{13}NO_3$	160.0968	160.0972	1.058-1.24
N-Acetyl-Alanine	$C_5H_9NO_3$	132.0655	132.0655	1.106-1.437
N-Acetyl-Aspartic Acid	$C_6H_9NO_5$	176.0553	176.0551	1.087-1.401
N-Acetyl-Glutamic Acid	$C_7H_{11}NO_5$	190.071	190.071	1.084-1.464
N-Acetyl-Glycine	$C_4H_7NO_3$	118.0499	118.0497	1.115-1.479
N-Acetyl-α-Lysine	$C_8H_{16}N_2O_3$	189.1234	189.1241	11.8-12.213
N-Acetyl-ε-Lysine	$C_8H_{16}N_2O_3$	189.1234	189.1236	10.286-10.799

Supplementary Table 3 - Acetylated amino acids chemical formulae, retention time, theoretical and experimental mass

Salmonella strains

Name	Description	Source or Reference
wild-type	12023 *S.* Typhimurium wild-type	NCTC
Δ*tacAT*	12023 Δ*tacAT::kan*(STM4401-4402)	(11)
Δ*tacAT2*	12023 Δ*tacAT2::kan*(STM5191-5192)	(11)
Δ*tacAT3*	12023 Δ*tacAT3::kan*(STM3505-3506)	(11)
Δ*tacAT*Δ*tacAT2*Δ*tacAT3*	12023 Δ*tacAT* Δ*taAT2* Δ*taAT3* unmarked	This work
Δ*parDE*	12023 Δ*parDE* unmarked (STM3562-3563)	This work
	SL1344	NCTC
	D23580	See Supplementary Table 1
	D25352	See Supplementary Table 1
	D23005	See Supplementary Table 1
	NCTC13349	NCTC
	D24954	See Supplementary Table 1
	D24793	See Supplementary Table 1
	D24359	See Supplementary Table 1

E. coli strains

Name	Description	Source or Reference
PC2	BL21(DE3)*endA*::Tet^R T1^R pLysS	(24)
BL21-AI	F-*ompT**gal dcm ara*B::T7RNAP-*tet*A	Invitrogen

Plasmids

Name	Description	Source or Reference
pBAD33	p15 *bla araC* P_{BAD}	This work
pBAD33::*tacT1*	pBAD33 P_{BAD} ::*tacT1*	This work
pBAD33::*tacT2*$_{STm}$	pBAD33 P_{BAD} ::*tacT2*$_{STm}$	This work
pBAD33::*tacT2*$_{SEn}$	pBAD33 P_{BAD} ::*tacT2*$_{SEn}$	This work
pBAD33::*tacT3*	pBAD33 P_{BAD} ::*tacT3*	This work
pBAD33::*parE*	pBAD33 PBAD::*parE*	This work
pBAD33::*tacT2*$^{Y137F}_{SEn}$	pBAD33 P_{BAD} ::*tacT2*$^{Y137F}_{SEn}$	This work
pBAD33::*tacT3*^{Y143F}	pBAD33 P_{BAD} ::*tacT3*^{Y143F}	This work
pBAD33::*tacT2*$^{R88G}_{SEn}$	pBAD33 P_{BAD} ::*tacT2*$^{R88G}_{SEn}$	This work
pBAD33::*tacT3*^{R94E}	pBAD33 P_{BAD} ::*tacT3*^{R94E}	This work
pCA24N::*tacA2*	pCA24N P*lac*::*tacA2*	This work
pCA24N::*tacA3*	pCA24N P*lac*::*tacA3*	This work
pBAD33::*tacT1*^{K31E}	pBAD33 P_{BAD} ::*tacT1*	This work
pRSFduet::*tacTA*	pRSFduet-1, *kan*, $lacI^{Q}$, T7::6His-TacT, T7::TacA	This work
pRSFduet::*tacTA2*$_{STm}$	pRSFduet-1, *kan*, $lacI^{Q}$, T7::6His-TacT2$_{STm}$, T7::TacA2	This work
pRSFduet::*tacTA2*$_{SEn}$	pRSFduet-1, *kan*, $lacI^{Q}$, T7::6His-TacT2$_{STm}$, T7::TacA2	This work
pRSFduet::*tacTA3*	pRSFduet-1, *kan*, $lacI^{Q}$, T7::6His-TacT3, T7::TacA3	This work
pQlinkH::TacT2$_{SEn}$^{Y137F}	pQlinkH, *bla*, $lacI^{Q}$, T7::7His-TacT2$_{SEn}$^{Y137F}	(22), This work
pQlinkH::TacT2$_{STm}$^{Y137F}	pQlinkH, *bla*, $lacI^{Q}$, T7::7His-TacT2$_{STm}$^{Y137F}	(22), This work

Supplementary Table 4 - Strains and plasmids used in this study
