# Supplementary material for: Activity of acetyltransferase toxins involved in Salmonella persister formation during macrophage infection
Source: Nat Commun. 2018 May 18;9:1993. doi: 10.1038/s41467-018-04472-6 (PMC5959882; doi:10.1038/s41467-018-04472-6)
Supplement: Supplementary file 2 — Description of Additional Supplementary Files [file 41467_2018_4472_MOESM2_ESM.pdf]

Description of Additional Supplementary Files

File Name: Supplementary Data 1
Description: Raw data of the LC-MS detection of acetylated amino acids after exposure to the different purified toxins.
